# Undergraduate Students’ Critical Online Reasoning—Process Mining Analysis

**DOI:** 10.3389/fpsyg.2020.576273

**Published:** 2020-11-30

**Authors:** Susanne Schmidt, Olga Zlatkin-Troitschanskaia, Jochen Roeper, Verena Klose, Maruschka Weber, Ann-Kathrin Bültmann, Sebastian Brückner

**Affiliations:** ^1^Department of Business and Economics Education, Johannes Gutenberg University, Mainz, Germany; ^2^Department of Neurophysiology, University Hospital of the Goethe University, Frankfurt, Germany

**Keywords:** online information processing, Critical Online Reasoning Assessment, person-oriented approach, event logs, eye-tracking, process mining, latent class analysis, repsonse process patterns

## Abstract

To successfully learn using open Internet resources, students must be able to *critically search, evaluate and select online information*, and *verify sources.* Defined as critical online reasoning (COR), this construct is operationalized on two levels in our study: (1) the *student level* using the newly developed Critical Online Reasoning Assessment (CORA), and (2) the *online information processing level* using event log data, including gaze durations and fixations. The written responses of 32 students for one CORA task were scored by three independent raters. The resulting score was operationalized as “task performance,” whereas the gaze fixations and durations were defined as indicators of “process performance.” Following a person-oriented approach, we conducted a process mining (PM) analysis, as well as a latent class analysis (LCA) to test whether—following the dual-process theory—the undergraduates could be distinguished into two groups based on both their process and task performance. Using PM, the process performance of all 32 students was visualized and compared, indicating two distinct response process patterns. One group of students (11), defined as “strategic information processers,” processed online information more comprehensively, as well as more efficiently, which was also reflected in their higher task scores. In contrast, the distributions of the process performance variables for the other group (21), defined as “avoidance information processers,” indicated a poorer process performance, which was also reflected in their lower task scores. In the LCA, where two student groups were empirically distinguished by combining the process performance indicators and the task score as a joint discriminant criterion, we confirmed these two COR profiles, which were reflected in high vs. low process and task performances. The estimated parameters indicated that high-performing students were significantly more efficient at conducting strategic information processing, as reflected in their higher process performance. These findings are so far based on quantitative analyses using event log data. To enable a more differentiated analysis of students’ visual attention dynamics, more in-depth qualitative research of the identified student profiles in terms of COR will be required.

## Introduction

### Study Background

The Internet and social media are among the most frequently used sources of information today. University students also often prefer online information to more traditional teaching materials such as textbooks (Brooks, 2016; List and Alexander, 2017; McGrew et al., 2019; Maurer et al., 2020). Learning by using freely accessible online resources offers many opportunities, but it also poses novel challenges. Conventional teaching material provided by universities is usually carefully curated by experts and tailored to maximize learning for students at clearly defined stages of their respective curricula. In contrast, the difficulty in using online resources lies in having not only to find suitable resources but also evaluate them without expert guidance. While the information stated on these websites may be correct and may simply not be well suited for the students’ learning aims, there are also more problematic scenarios. It has become evident that not only “fake news” but also “fake science” characterized by scientifically incorrect information circulates on the Internet (Ciampaglia, 2018). Therefore, to successfully learn by using open Internet resources, students must be able to critically search, evaluate, select, and verify online information and sources. In addition to a critically minded attitude, students need the ability to distinguish reliable from unreliable or manipulative information and to question and critically examine online information so they can build their knowledge and expertise on reliable information.

In recent years, however, there has been increasing evidence that university students struggle to critically assess and evaluate information from the Internet and are often influenced by unreliable sources (McGrew et al., 2018; Wineburg et al., 2018). Although current research assumed that search context and strategies were related to the information seeking and evaluation processes, not much is known about the specific search strategies and activities of students on the web, especially with regard to learning using the Internet (Walraven et al., 2009; Collins-Thompson et al., 2016).

To investigate how university students deal with online information and what influences their information processing, we used the newly developed online test “Critical Online Reasoning Assessment” (CORA), which is based on the Civic Online Reasoning assessment developed by the Stanford History Education Group (Wineburg et al., 2016). During the assessment, the test takers are presented with novel performance tasks; while working, they are asked to freely browse the Internet to find and select reliable information relevant for solving the given tasks within the relatively short time frame of 10 min. As part of their written response, they are asked to justify their task solutions by using arguments based on their online research. For the study presented here, university students from three disciplines (medicine, economics, and education) at two universities in two German federal states were tested using CORA (for details, see the section “Study Design”).

Recently, the Standards for Educational and Psychological Testing (American Educational Research Association [AERA] et al., 2014) have emphasized the particular importance of test “validity evidence’ based on response processes. Response processes refer to psychological operations, approaches and behaviors of test takers when they carry out tasks and create solutions. They are revealed through response process data, i.e., verbalizations, eye movements, response times, or computer clicks (Ercikan and Pellegrino, 2017). Therefore, American Educational Research Association [AERA] et al. (2014) introduced the response processes as one of the central criteria of “validity,” i.e., “the degree to which a test score can be interpreted as representing the intended underlying construct” (Cook and Beckman, 2006). These processes need to be distinguished from construct-irrelevant processes, i.e., not defined by the construct (e.g., guessing as a task processing strategy).

Following this paradigm in educational assessment, which focuses on validation of scores by means of analyzing response processes (Ercikan and Pellegrino, 2017), the aim of this study was to obtain validity evidence about response processes. This entails (i) describing the processes underlying students’ critical online reasoning (COR) when solving the CORA tasks, as well as (ii) analyzing the relationship between the task scores (“critical online reasoning”) and the response processes of the CORA participants to define the extent to which the response processes typically reflect information processing and solving strategies associated with the COR construct (see the section “Construct Definition and Fundamental Assumptions”).

### Research Objectives

As recent research on online information processing indicated (see the section “Theoretical Framework”), web searches entail cognitive and metacognitive processes influenced by individual differences. Therefore, students with similar cognitive and/or metacognitive abilities tend to develop very different search and information processing strategies (e.g., Kao et al., 2008; Zhou and Ren, 2016; Brand-Gruwel et al., 2017). This is particularly true for CORA, where students’ spontaneous web searches in a natural online environment were measured *in vivo*. To precisely describe and analyze the different patterns in students’ CORA task solution processes and online information processing, we introduced a new method of process mining (PM).

To comprehensively examine students’ response processes in detail when dealing with online information, their web search activity while solving the CORA tasks was recorded for analysis. The collected data included (i) the entire log data collected throughout the task-solving process, including all websites the students accessed during their search, as well as the eye movements of test takers recorded during their CORA task processing and online search to gain additional insights into the participants’ cognitive processes while using online media; and (ii) the videos of their task-solving behavior recorded by the eye tracker, to more directly observe what students do and do not do while solving CORA tasks (for details, see the section “Study Design”).

Starting from a theoretical process model (see the section “Theoretical Framework”), the main research objective of this article is (i) to describe the response processes of students dealing with online information while they are working on the CORA tasks, and (ii) to investigate the relationship between the students’ online information processing strategies and their performance on the CORA tasks, in particular, by comparing students with high vs. low process and task performance.

Based on event logs including eye-tracking (ET) data (see the section “Materials and Methods”), as indicators of students’ response processes, log files and eye movements while solving the CORA tasks were considered. This provided information about the numbers, types, and orders of the main process steps (such as reading the instruction, Google searches, selecting websites, reading webpages, writing response), and the durations and fixations per individual process step (for details, see the section “Materials and Methods”).

Using the PM approach, we investigated the following research questions (RQs):

*RQ1:* How can the students’ response processes, related to their COR while solving CORA tasks, be visualized and precisely described?

*RQ2*: What are similar and distinct patterns in the students’ response processes related to their COR based on

• their process performance variables: fixations per individual process steps as well as the duration, number, type, and order of the individual process steps?
• how are they related to the (overall) task performance, i.e., the score of the written responses?

To answer these RQs, the log file data using ET data were prepared, and patterns contained in event logs recorded in the eye tracker while the participants solved the CORA tasks were identified and analyzed using PM (see the sections “Study Design” and “Materials and Methods”). In the next step, the raw ET and log data were processed and transformed into a newly generated and aggregated data set, which was used in the subsequent model-based statistical analyses [latent class analyses (LCA)] to test the research hypotheses (see the section “Results”).

## Theoretical Framework

### State of Research and Conceptual Foundation

There is currently no unified framework for and definition of COR in the literature. Existing studies about students’ information-seeking behavior (Hargittai et al., 2010) and the underlying processes (e.g., “search as learning,” Hoppe et al., 2018) have been based on different frameworks and research traditions: recent research on *online* information processing and behavior was primarily based on frameworks developed in the context of “multiple source use/comprehension” (e.g., “multiple documents literacy,” Anmarkrud et al., 2014; for an overview on MSU, see Braasch et al., 2018) and “information problem solving” (Brand-Gruwel et al., 2005). These approaches required a decomposition of different (meta-)cognitive subskills and solving strategies such as “defining the problem” to deal with information problems. One research strand has been particularly focused on the credibility of web-based information as an explanatory factor of online information-seeking behavior (e.g., approaches on “web credibility,” Fogg et al., 2003; Metzger and Flanagin, 2015; “credibility evaluation,” Metzger et al., 2010; “information trust,” Lucassen and Schraagen, 2011). Recent studies indicate the particular role and influence of cognitive heuristics that information users employ when evaluating the credibility of online information and sources. Moreover, credibility evaluations appear to be primarily due to website characteristics.

With regard to “reasoning,” the research refers to well-established traditions, focusing in particular on generic reasoning and scientific reasoning (e.g., Fischer et al., 2014; Stanovich, 2016) and (corresponding) reasoning biases and heuristics (Kahneman et al., 1982; Gigerenzer, 2008; Hilbert, 2012) of information processing and decision making (for an overview, see, e.g., Stanovich, 2003; Toplak et al., 2007). More specifically, for instance, recent results indicate that most students routinely applied (meta-)cognitive heuristics (e.g., self-confirmation heuristics) to process and to evaluate the credibility of online information and sources (Metzger et al., 2010).

Research with a particular focus on students’ web search behavior and their navigation of online environments and online information resources, however, is still relatively scarce. The existing studies focusing on process analysis of students’ use of multiple online sources and information, which we described as “online reasoning” (McGrew et al., 2019), are based on an amalgamation of several theories and frameworks of cognition and learning; in particular: (i) development of expertise in (meta-)cognitive and affective information processing with different media (Mayer, 2002; Alexander, 2003; List and Alexander, 2019); and (ii) critical use of multimodally represented information from multiple sources (Shaw, 1996; Wiley et al., 2009; Braasch and Bråten, 2017; List and Alexander, 2017; Yu et al., 2018). For instance, List and Alexander (2017) found four different navigation profiles of satisfying approaches when students navigate information without time limitations (e.g., the limited navigation profile, and the distributed navigation profile); these profiles were differently correlated with task performance.

Over the last few years, process research on (online) learning behavior (Ercikan and Pellegrino, 2017; Zumbo and Hubley, 2017) using verbal data (Leighton, 2017) and computer-generated data (Goldhammer and Zehner, 2017; Li et al., 2017; Oranje et al., 2017; Russell and Huber, 2017) (e.g., log data, ET, dwell times) has increasingly been developed to gain insights into students’ (meta-)cognitive information processing, indicating that response process evidence (“cognitive validity”) is required to validate claims regarding the cognitive complexity of performance assessments such as CORA.

### Construct Definition and Fundamental Assumptions

When developing the conceptual framework for this study, we used the broad definition of COR, which describes the ability to effectively search, verify (i.e., to prove the accuracy), and evaluate (i.e., to draw conclusions from examining) online information (McGrew et al., 2018), as a starting point. We claim, therefore, that COR expresses itself in the ability to identify the author and/or the organization behind a source of information and to make an informed assessment regarding their motives and their trustworthiness, to verify their claims by consulting other (reliable) sources, and finally to come to a conclusive decision about the utility of the source.

To capture the response processes underlying COR, the construct is further defined by including more distinct facets of online information processing and information problem-solving strategies. First, the individual’s distinct phases within the online information processing are described in more detail. For systematizing and classifying these processes, we applied a descriptive approach (e.g., Gerjets et al., 2011) based on the Information Problem-Solving (IPS-I) model (Brand-Gruwel and Stadtler, 2011). This model distinguishes constitutive basic abilities of problem solving when using online information, which are activated by regulatory and conditional skills, for instance, searching, scanning, processing, and evaluating (online) information.

Second, to qualitatively describe how these skills manifest, the subprocesses involved in processing online information are classified into heuristic and systematic processes in accordance with the dual-process theory (Chen and Chaiken, 1999; for the heuristic analytic process model, see Evans, 2003). This theory has already been established in several research domains, including inference and reasoning (De Neys, 2006; Evans, 2006), the evaluation of credibility (Metzger et al., 2010), as well as in the context of ET studies (e.g., Horstmann et al., 2009; Wu et al., 2019). Horstmann et al. (2019) claimed, for instance, a relation between (meta-)cognitive heuristics and the different components of visual search such as skipping (distracters), dwelling, and revisiting.

Originally, this theory was developed to explain the prevalence of cognitive bias in argumentation tasks (Evans, 1984, 1989). According to different research perspectives, heuristic and systematic cognitive processes can occur either simultaneously (“Parallel Models,” Sloman, 1996) or sequentially (“Default Intervention Models,” Evans and Stanovich, 2013). Heuristic processes are mostly experience-based (Gronchi and Giovannelli, 2018) and are assumed to occur fast, unconsciously, automatically, and with low cognitive effort (Horstmann et al., 2009; for more details about heuristics, see Stanovich, 2012; for schema theory, see Anderson et al., 1978). Systematic processes require a higher cognitive effort, make use of complex mental models, and activate deliberative reasoning; they are goal- and rule-driven, analytical, precise, and based on weighing up the positive and negative aspects of various options (Chen and Chaiken, 1999; Evans and Stanovich, 2013).

The existing studies that focus on the heuristic and systematic processes of information seeking indicate the common use of cognitive heuristics instead of deliberate strategies while evaluating and comparing online information (Horstmann et al., 2009). The perceived ranking of web search results has an important impact on the evaluation and judgment of online information (Hargittai et al., 2010). For instance, students rely immensely on the ranking provided by search engines and mostly access only the first few websites presented (e.g., Walraven et al., 2009; Gerjets et al., 2011; Zhang et al., 2011). Observing domain experts while solving an information problem and comparing them to novices, Brand-Gruwel et al. (2009) showed that experts spend more time on the process steps of information problem solving, and they metacognitively evaluated their solving process more often. Experts were also more likely to alternate between searching and viewing webpages and decided to leave webpages to return to the hit list faster than novices. Collins-Thompson et al. (2016) showed that the amount of time web searchers spend on one document during the searching process was positively correlated with their higher-level cognitive learning scores. Likewise, Anmarkrud et al. (2014) indicated the positive relationship of students strategic processing while reading the documents and source awareness in multiple document use of undergraduates. Wineburg and McGrew (2017) found that professional “fact checkers” read laterally and leave a website after a quick scan to initially gain more insight into the credibility of the website through external sources, whereas less experienced students read vertically and judge the website according to its own attributes.

Based on prior research, we assume that COR should be based on strategic information processing, i.e., a combination of both experience-based (meta-)cognitive heuristics to efficiently process online information, which can be applied flexibly in the context of information problem-solving in certain (task-related) situations, as well as systematic processes to activate the deliberative (“critical”) reasoning; strategic information processing may relate to a better “process performance” and “task performance.”

### Eye Movements as an Indicator of Cognitive Processes

To provide insights into students’ cognitive information processing, in particular, operationalized through gaze durations and fixations, ET is increasingly used in related research (Horstmann et al., 2009; Gerjets et al., 2011). Accordingly, sequences of eye movements are identified that can be used to operationalize students’ depth of processing and thus draw inferences on their cognitive effort. The focus of cognitive information processing is generally on attention-related processes (Orquin and Loose, 2013). Therefore, fixations on so-called “areas of interest” (AOIs) are often chosen as an indicator of cognitive effort (Gerjets et al., 2011; Raney et al., 2014). Fixations are periods of stabilized eye positioning (fixed gaze), during which a small AOI in the visual field (about the size of the moon in the sky) is presented on the fovea, the part of the retina with the highest visual acuity (Duchowski, 2007). Complex visual scenes are analyzed by a sequence of fixations under attentional control and are separated by jump-like eye movements every few hundred milliseconds (saccades). Given that fixations give access to highly resolved visual information, they are also indicators of cognitive processing depth (Holmqvist et al., 2011) and might therefore be considered as surrogate markers for “process performance.” Thus, a basic assumption of ET is that increased processing demands are associated with increased processing time and/or changes in the patterns of fixations. Increased processing time may be reflected by longer fixations and/or larger numbers of fixations (forward and regressive) (Raney et al., 2014). For instance, in an ET study, Zhou and Ren (2016) examined the cognitive strategies of students, focusing on their searching process when looking for specific online information. They found that high-performing students revised search queries more often and spent more time reading and assessing the information in the selected webpages for its relevance. Moreover, high performers switched more frequently between search results and webpages before staying on a certain webpage, which might indicate a more critical metacognitive engagement.

As eye movements are influenced by numerous factors (e.g., Reitbauer, 2008; Cyr and Head, 2013), conclusions from ET data on information processing and online reasoning—in accordance with the dual process theory—constitute a model-based method of complexity reduction to gain first insights into these processes and their relationships with students’ CORA task performance. We assume that students’ eye movements while solving CORA tasks reflect their process performance. More specifically, fixations per individual process step, as well as the duration, number, type, and order of the individual process steps—in accordance with the IPS Model—are used as indicators of students’ process performance (see the section “Materials and Methods”) and are related to their task performance (i.e., the score of the written responses). In addition, following the dual process theory and the aforementioned findings from related ET research, we assume two distinct patterns in the students’ process performance related to their COR.

## Study Design

Critical online reasoning was measured using the newly developed CORA (Molerov et al., 2019). Originally, it contained five tasks, which we reduced to two representative tasks for this PM study, i.e., analysis of log events based on ET data. In the tasks, participants were presented with URLs that directed them to a website (published by a company focusing on online marketing) about vegan protein sources (Task 1) and a tweet (published by a market-liberal organization) about German state revenue (Task 2), respectively. These sources of online information were to be evaluated by the participants with regard to credibility in a free-answer format. They were also asked to give evidence by providing URLs to websites/sources that supported the argumentation in their task response (written statement).

The two CORA tasks were implemented into an ET test environment using Tobii Pro Lab software and hardware. To facilitate the subsequent extraction and comparisons of the test participants’ data, especially with regard to how they used the websites linked in the URLs, a web stimulus presentation was used instead of a more open screen recording. To integrate the two tasks into a web stimulus, an online writing document was generated using the web-based text editor EduPad (EduPad is a collaborative text environment based on the Etherpad software^1^). Participants were asked to formulate their argumentative task response in the same EduPad document (hereafter referred to as “Task Editor”). During the CORA, they could use any information on the provided website/tweet and were also asked to use other websites and search engines, to stimulate a naturalistic online information processing and problem-solving behavior. The students could switch between websites and the document containing the tasks and reread the task prompt or edit their written task response.

Participants were given 10 min per task to complete the two CORA tasks (excluding the time required for calibrating the eye tracker). A short instruction (hereafter referred to as “Reading Instruction”) at the beginning of the task informed the participants about the study procedure as well as about suggested approaches to solving the task, i.e., using all information on the linked websites as well as other websites, using search engines, and how they could return to the task editor. After reading the instruction, students had to actively start the task by activating the task editor EduPad.

Process data, which comprise log events including eye movements during the CORA, were recorded with a Tobii Pro X3-120 eye tracker using a sampling rate of 120 Hz. The recorded data provided further details about participants’ response processes and how they processed and interacted with information online, for instance, through mouse clicks, query streams, or weblogs. To visualize the information processing behavior of each student, we used PM, where fixations were counted for all task process steps, including for every single webpage. Thus, the sum of fixations for each webpage was calculated and considered an additional indicator of the participants’ process performance while working on the CORA tasks (for details, see the section “Data Transformation”).

After the CORA, raw event log data that contained the fixations and web search events of all participants at the millisecond level were exported, prepared, and transformed to exploratively determine the response process steps and students “process performance” using a PM approach (see the section “Process Mining”). Based on the PM analysis, we gained insights into the process steps as well as the distinct process steps of each student. “Distinct,” for the purposes of this study, referred to the sum of identical (but potentially repeatedly executed) process steps. For instance, if a student opened a website, this was probably followed by some activity on that particular website, like reading or scrolling, and then the student would typically return to the task editor, to take, for instance, several notes about the contents on this webpage or to copy the hyperlink as a reference for the written response. However, the student then may have returned to that same website, which the event log would record as a next process step with the same name but with a different timestamp. Therefore, the event log data list at least three process steps (website → “Task Editor” → website), but the number for *distinct* activities would be only two as the student repeated one unique process step (visiting the same website twice).

This transformed data were then used for exploratory PM analyses. as well as statistical model–based analyses to test the formulated hypotheses (see the section “Discussion of Process Mining Results and Research Hypotheses”). The process data were also aggregated (such as the average number of fixations per process step of each student) and combined with the “task performance” data, which included the dependent variable of the score of the students’ written responses to the CORA tasks. The students’ responses (written statements) were scored by three independent (trained) raters using a developed and validated rating scheme ranging from 0 to a maximum of 2 points (as a 5-point Likert scale: 0, 0.5, 1, 1.5, 2; for details, see Molerov et al., 2019).

Based on a person-oriented analysis approach, the hypotheses on the expected significant differences between the students in terms of their “process performance,” which was calculated based on the (i) assessed and aggregated process-related variables using the number, of total process steps as well as the number of distinct processes performed, the average number of fixations as well as the average duration of each process step for every student, all of which were analyzed in relation to (ii) the students’ task performance (CORA task score), were investigated by means of an LCA (see the section “Latent Class Analyses”).

### Sample

In total, 32 undergraduate students from the fields of medicine (9 participants), economics (9 participants), and business and economics education (14 participants) took part in the CORA. In the context of three obligatory lectures or seminars (in economics, education, and medicine) at two German universities, all students attending these courses were asked to complete a paper–pencil survey to assess their domain-specific knowledge and other personal characteristics (e.g., fluid intelligence, media use). Subsequently, the students who had participated in this survey were asked to take part in the CORA as well, to recruit approximately 10 test persons from each of the three groups, in accordance with the purposeful sampling method (for details, see Palinkas et al., 2015).

For this article, a purposefully selected sample of participants was used, as the amount of data for an unselected number of participants would have been too large and not feasible for practical research purposes. When selecting this sample, we included students from all study semesters, and we selected students from two disciplines to initially control for domain specificity. Another important criterion for the sampling was the students’ central descriptive characteristics such as gender, age, migration background, and prior education, which may influence their web search behavior and COR task performance.

For the first task, the data on all 32 participants could be used, whereas for the second CORA task, the sample was reduced to *N* = 30, as the survey had to be terminated prematurely because of technical problems for two participants. Additional background information on the participants, such as gender, age, and study semester, was assessed. The average age for the sample was 22.37 years (SD = 4.1 years); 71% of the 32 participants were female. Most of the participants were in their first or second year of study. With regard to the distribution of the descriptive characteristics such as age and gender, no significant deviations from the overall student population in these study domains were found. As participation in this study was voluntary, however, a bias in the sampling cannot be ruled out (see the section “Limitations”).

## Materials and Methods

### Process Mining

A unique aspect of the CORA is that the assessment of students’ spontaneous web searches took place in a naturalistic online environment, with a freely accessible world wide web without any restrictions (besides a limited task processing time of 10 min). At the same time, this makes data analysis particularly demanding and requires a precise and differentiated description of the data in its sequential process structure in the first step. To be able to answer the two RQs, RQ1, and RQ2, which aim to visualize, describe, and discover patterns in the students’ CORA task response behavior, we focused on the method of PM to analyze process-related student data as we tracked in detail how each student approached the CORA task. For PM data transformation and visualization, we used PAFnow^2^.

Process mining is an aggregative, visualization methodology to gain insights and acquire knowledge about the test-taking process behavior of individual participants, recorded in event logs. In educational research, this is typically based on data collected in computer-based assessments (Tóth et al., 2017). Using the visualized data collected through PM and representing students’ processing behavior in a process graph can reveal information about the homogeneity and heterogeneity of students’ “process performance” (which can then be related to their task performance in the next step, see the section “Latent Class Analyses”). By comparing the *number* and *kind* of process steps, the *order* of these process steps or the time spent on each process step, i.e., duration, we explored whether there were similarities or differences (at the process level) between or within students.

In the present study, the number of fixations within each process step was also included in the analyses. Hereby, we distinguished between the analyses at the process level or at the student level. The number of process steps, for instance, related to the student level. The number of fixations, however, can also refer to the process level, as these data were recorded for every single activity of the participants on a millisecond level and with high temporal resolution. For the PM analysis, the number of fixations was aggregated for each meaningful process step. Accordingly, the conducted analyses referred to different levels: the PM approach refers to the process level, whereas the LCA refers to the student level (according to a person-oriented approach, see the following section).

### Person-Oriented Approach and Latent Class Analyses

To investigate RQ2, the additional methodological focus for the analyses of students’ response process data was—in accordance with Bergman and El-Khouri (2001)—on the person-oriented approach, which states “that interindividual differences and group differences need not be added to the error variance, and that they are worthy of being made the object of investigation” (von Eye, 2006, pp. 11–12). As prior research on (online) information processing indicated, the way information is processed—in our case, while solving the CORA task—is (at least partially) (sub)group-specific (Kao et al., 2008; Zhou and Ren, 2016; Brand-Gruwel et al., 2017). Given this assumption (see the section “Construct Definition and Fundamental Assumptions”), we aim to empirically uncover the existence of possible subgroups in the student population, i.e., to investigate latent subgroups. The term “subgroups” is defined as a set of participants in the sample who are more similar to each other in terms of task and process performance than others. The division of the student population into subgroups focuses on the individual differences between the students, as these differences have a decisive influence on and characterize the information processing (Sterba and Bauer, 2010). These group differences or the affiliation of the participants to certain subgroups was not known *a priori* in the present study. This was taken into account in our study by examining whether the students were divided into latent subgroups based on their task score and the indicators of their process performance, i.e., the duration of the distinct process steps, the number of performed (distinct) process steps, and the number of average fixations per process step. The person-oriented approach allowed for an adequate testing of the research hypotheses against the backdrop of strong heterogeneity and different information processing approaches of the participants.

Based on the “dual process” model (see the section “Construct Definition and Fundamental Assumptions”), it was assumed that depending on both their task performance as well as on their process performance, the students population can be empirically divided into two subgroups: (1) high performers and (2) low performers. It was supposed that the process performance not only leads to an according test performance (CORA task score), but it is also reflected in process performance indicators that can provide a comprehensive picture about the cognitive information processing strategies the students used to achieve a (higher) test score, such as duration, fixations, and number of (distinct) process steps. Therefore, the investigation of high and low performers not only accounted for the task score, but also for process-relevant variables, using an LCA as an appropriate empirical model to test whether the supposed multigroup structure can be empirically determined.

The probability that a person *s* has a certain response pattern is the same for all persons; therefore, $p\,\left(a_{s}\right)=\prod_{b=1}^{5}p\,\left(y_{i_{b}}\right)$ (Rost and Eid, 2009, p. 490), with *i* as CaseId and *b* = {task score, total fixations, total duration, number of process steps, number of distinct process steps}. Taking into account the latent class belonging to one of the two classes *c1* (high performer) or *c2* (low performer), the results in the conditional probability are $p\,\left(a_{s}|c\right)=\prod_{b=1}^{5}p\,\left(y_{i_{b}}|c\right)$. These two response pattern probabilities are required to determine the conditional class belonging to probability *p*(*c*|*a*_*s*_). The goal of a model of LCA is therefore to predict the probability that a person *s* conditionally belongs to a certain class *c* based on his/her “process performance” vector *a*_*s*_. The model is as follows (Gollwitzer, 2012, p. 302):

$$p\,\left(c|a_{s}\right)=\pi_{c}\,\frac{p\,\left(a_{s}|c\right)}{p\,\left(a_{s}\right)},$$

where π_*c*_ = *p*(*c*) stands for the unconditional class belonging to probability (relative class size), with π_*c*1_+π_*c*2_ = 1 (i.e., each person in the sample belongs to exactly one latent class *c*). The unconditional “process performance” indicators can thus be defined as a discrete mixture of the conditional probabilities of performance patterns, and the following holds for both classes *c1* and *c2* (Rost and Eid, 2009, p. 490; Gollwitzer, 2012, p. 302; Masyn, 2013, p. 558):

$$p\,\left(a_{s}\right)=\pi_{c\,1}\cdot p\,\left(a_{s}|c\,1\right)+\pi_{c\,2}\cdot p\,\left(a_{s}|c\,2\right).$$

The LCA was conducted using Stata 16^3^ with an identity link and reporting the conditional classification for each student and the predicted category of being a high or low performer. The global LCA model fit was evaluated using Akaike’s information criterion (AIC) and Bayesian information criterion (BIC) where the two-class model was benchmarked versus a one-class model to test whether the hypothesis of having two groups in the sample (high- and low-performing students) can be confirmed (see the section “Latent Class Analyses”).

### Data Transformation

To conduct a PM analysis to visualize the response processes (RQ1) and to perform the LCA to investigate the latent subgroups based on their process performance indicators to find similar and distinct patterns in both their task performance as well as in their response processes (RQ2), process data are required. To collect the process data of each student while working on the CORA task, the eye-tracker Tobii X3-120 (120 Hz) was used. We gathered ET information on both gaze-related data, such as eye movements and fixations (e.g., on the webpages) and additional process-related data, such as the different events during the web search, including the URL of the visited website and the keyboard events. For instance, when the response to the CORA task was written, the data were recorded and stored by the eye tracker in a tsv-formatted table (for an example of the raw data, see Supplementary Table S1). The durations of these events were also documented.

The raw data from the eye tracker were calibrated on the milliseconds level: each key stroke and each gaze point were stored in the data set, so that eye movements were temporally aligned to other process data. For the PM approach to reproduce the process behavior of the web search and task performance, however, a higher time level is required. The following steps of data processing and aggregation were conducted to create a meaningful event log that allowed the interpretation of students’ response processes regarding the CORA task:

#### Evaluation of Event Occurrences and Analysis of Visited Websites

In the eye-tracker data set, three different types of events were recorded: keyboard events, mouse events, and URL events. Keyboard events comprised typical writing events, such as pressing letter or number keys, as well as other events, such as changing window ([alt] + [tab]) or copy/pasting events ([ctrl] + c/[ctrl] + v). Mouse events are typically just scrolling or clicking activities. Regarding URL events, each and every single URL that was accessed by a student was recorded (see Supplementary Table S2).

#### Aggregation of Single Events to Distinct Process Steps

Based on the findings from the analyses of visited websites (incl. webpages), we aggregated these results for occurrences between students. For defined typical events, such as watching a YouTube video, conducting a web search using Google, gathering information from a newspaper website or other common URLs, the occurrences were translated into a comparable process step. If student A opened YouTube and watched a video X and student B watched video Y, the differences between the videos in terms of content were not further considered as it was not relevant for the PM analysis. This analysis does not aim to explain the differences between students who opened different YouTube videos or who gathered information from different newspapers. Therefore, the commonly visited URLs’ events were aggregated in the meaningful categories, and then into (distinct) process steps. URLs that were visited by only very few students were not aggregated (for a detailed overview of the URLs’ events and their aggregation to process steps, see Supplementary Table S2).

Moreover, keyboard events were also aggregated using a similar procedure as described in (1). All activities related to writing were summarized under “Keyboard Event—Writing” and all other keyboard events were summarized under “Keyboard Events—Other.” Similarly, all mouse events were also aggregated in one process steps. In addition to URL events, mouse and keyboard events, the process steps of reading the instructions (welcome text and general description of CORA), and task editor (where the CORA task itself is shown and where the students write their responses) were also distinguished. In addition, the event log also consisted of further (less informative but still process-relevant) process steps, such as “Eye-Tracker Calibration,” “Recording Start and End,” or “Web Stimulus Start and End” (for the full event log, see Supplementary Table S3).

For the PM analyses, we focused only on the most important process steps to visualize the construct-relevant web search and task-solving process (for an example of a shortened event log for the PM analyses, see Supplementary Table S4). For instance, mouse events were excluded from the PM analysis, as they did occur at any time and were associated with most processes (e.g., while reading the instructions, during web searches, or while writing the response).

#### Summarization of Fixations and Working Time for the Particular Process Steps, as Indicators of “Process Performance”

For all process steps described in 2, duration, i.e., time spent on each process step as well as the number of fixations recorded in each process step, was calculated. When aggregating the raw data as described above, both indicators of “process performance,” the number of fixations and the duration, were summarized for each process step (for details, see the section “Process Mining”). Based on this strategy, a comparison of duration and fixations of each student and for each single process step was possible, as well as a comparison of the same “process performance” indicators (such as duration and the number of fixations) between different students.

#### Building a Data Model With Process (Event Log Table) and Student (Case Attributes Table) Related Variables

To conduct PM analyses and investigate process behavior on the process level, a data set with process-related variables was required in which the sequence of the process steps was in the correct temporal order. This table was called an event log table, as it comprises all relevant variables on the event level. An event log showed the unique identifier for each student (CaseId) and the process steps each student executed, as well as the according timestamps. Furthermore, the event log consisted of the number of fixations for each process step (for an example of an event log of one student, see Supplementary Table S3).

To explain “process performance” between students, variables on the student level, such as the CORA task score, were required. Based on the unique student identifier, the CaseId, a separate table for process-related variables aggregated on the student level, was added to the data model. This table was called case attributes table and consisted of the CORA task score and all aggregated process variables for each student: the total number of fixations and the total time spent on the task (total visit duration), as well as the total number of process steps (e.g., the count of the rows of the event log table as aggregated process steps, see Supplementary Table S4) and the number of distinct process steps. For the latter, for instance, while the total number of process steps for one student was 30, within these 30 steps, he/she read the instructions, opened the task editor, and immediately started to *write* the response, so that the number of distinct process steps would only be three in this case.

Based on these four major steps regarding data preparation, a new transformed and aggregated data set was created that allowed for the following analyses as described in the section “Results.”

## Results

### Process Mining

Using PM as an explorative approach to visualize and precisely describe the students’ response processes while solving the CORA tasks (RQ1), first, the processes students applied while working on the CORA tasks were analyzed for the entire sample of 32 students. The process steps while working on CORA task 1, which included an unrestricted web search, were visualized and analyzed by first comparing the structure of the entire process graph for all students, including all events that were recorded by the eye tracker (see the section “Data Transformation”), to evaluate the students’ task-solving behavior and process performance. The aim was to reveal potential common patterns in the students’ performance variables, such as fixations, duration, and process steps (RQ2), which go along with the COR construct definition of searching, evaluating, and refining information before or while formulating a response (see the section “State of Research and Conceptual Foundation”). However, a visualization of all the process steps in one process graph did not provide the type of information that would have allowed for meaningful interpretation and for answering the two RQs, as a precise revelation of distinct or common patterns in the students’ response processes was hardly possible (see Supplementary Figure S1). For the following PM analysis, only the process steps of “Reading Instruction,” “Task Editor,” as well as all URL events were included (for an example of short event log for PM analysis, see Supplementary Table S4), so that the visualization of the process graph was readable, and the focus was on the interpretation of the web search activities. “Keyboard Events” and “Mouse Events” were also excluded from the process graph, as they could occur at any time during each process.

**FIGURE 1:**
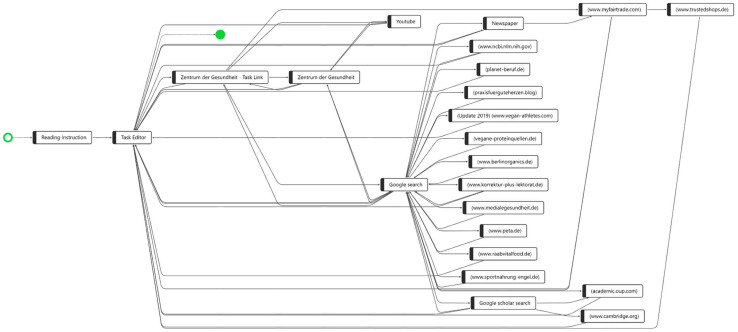
The process steps of all 32 participants while working on CORA task 1.

Figure 1 shows the process variants of all 32 students while working on CORA task 1 combined in one graph. The starting point (on the left side marked as a green hollow circle) has only one arrow pointing at the process step “Reading Instruction,” indicating that all students started by reading the instruction. In the second process step, all students opened the “Task Editor,” which had two functions in the computer-based assessment format used in the CORA: (1), it contained the task description as well as the task prompt, and (2) it was the text editor where the students wrote and submitted their responses to the CORA task (see the section “Study Design”).

The process sequence (visualized by the arrows in the graph) at the beginning of the CORA task was identical for all students of our sample: “Reading Instruction”? “Task Editor.” After the process step of opening the task editor and reading the task prompt, and perhaps already starting to write the response, however, the process graph became less clear. The arrowheads enable a differentiation between whether a sequence flows *from* the “Task Editor” to, for instance, the website linked in the CORA task “Zentrum der Gesundheit—Task Link” or back from any process step *to* the “Task Editor,” indicating that the sequences around the “Task Editor” to or from any other process step are manifold.

**FIGURE 2:**
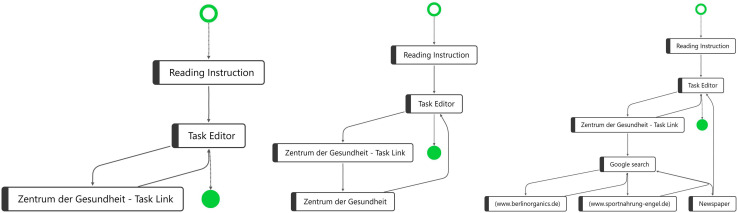
Three process graphs, with the most frequent variant **(Left)** in the sample, the second most frequent variant **(Middle)**, and one randomly chosen variant **(Right)**.

Another important visualization is the green, filled circle, which indicates the end point of task processing; i.e., the “Task Editor” is the only ending point in the process indicating that all students ended the task with writing their response. While performing a Google search appears to be a common behavior among the 32 participants, after the process step “Google search,” a total of 16 different websites were visited by the participants. Based on the visualization of the order of the sequences of the process steps for all students, however, we do not know whether these 16 websites were visited by all 32 students (or only by one), and we cannot yet identify any common patterns in this process graph. Between the starting point and the end point, the visualization of all 32 participants’ processes in one process graph does not allow for a precise description of the processes performed and, for instance, observing whether there is a common process variant regarding the complete task processing and where the distinct differences in information processing between the students are. Therefore, in the next step, the individual variants were explored separately, indicating that of 32 students, almost every student navigated the CORA task differently. However, we also managed to find some common patterns (Figure 2). In contrast to Figure 1, Figure 2 shows a top-to-bottom visualization of the process graph; i.e., the starting point is now at the top and the end point is at the bottom.

One frequent process variant, the process path for four of the students, is shown on the left side of Figure 2. Another frequent variant, applied by seven students, is shown in the middle of Figure 2. The difference between the two variants is that in variant 2, the students opened another webpage by “Zentrum der Gesundheit” instead of directly returning back to the “Task Editor.” This behavior can be defined as an “avoidance strategy,” as these seven students did not conduct a web search at all. They completely skipped searching and evaluating additional online information—two processes that are a crucial part of COR according to our construct definition (see the section “Construct Definition and Fundamental Assumptions”). In another variant, two students showed a similar behavior as in variant 2, but they additionally opened a YouTube video directly after visiting “Zentrum der Gesundheit” (see Supplementary Figure S2). Similarly, we found a variant in which three students, in addition to the behavior in variant 2, opened Google and entered a search term, but did not proceed to access a website; instead, they returned directly back to the task prompt (see Supplementary Figure S3). Following our COR construct definition, this behavior can also be interpreted as an “avoidance strategy,” as it cannot be assumed that these students applied critical reasoning to evaluate the trustworthiness of the task link.

Through this visualization, we identified that for 16 students (i.e., half of the sample), the response processes while working on the CORA task (despite the individual differences) can be classified as an “avoidance strategy.” At the same time, the explorative PM analysis revealed that there are also students who conducted a web search after opening the task prompt, as required by the COR framework. On the right side of Figure 2, a randomly chosen process for one single student—of the remaining 16 different response processes—is shown. This student who conducted a Google search opened three different websites: (a) www.berlinorganics.de, (b) www.sportnahrung-engel.de, and (c) a news website. Regarding this process behavior, we can assume that this student researched, verified, and refined the results before formulating her/his response. Exploring the process graphs for the other 15 students showed a similar process behavior, but with a variation in the number of distinct process steps. Some students opened only a few websites in addition to the website linked in the task prompt; others, like the student shown on the right side in Figure 2, performed several process steps and visited many websites. According to our assumption (see the section “Construct Definition and Fundamental Assumptions”), behavior of this kind among students in this group indicates a “strategic information processing strategy.”

In the next step, taking into consideration the results on task performance at the student level, we found with regard to the three variants shown in Figure 2 that the variants on the left and in the middle show students who employed “avoidance strategies” and mostly achieved scores between 0 and 0.5 on the task, whereas students who used a strategic information processing strategy achieved scores between 1 and 2 on the CORA task.

Overall, Figures 1 and 2 indicated that despite visualizing either all process variants or only the most frequent variants in the sample, it was still not possible to generate precise descriptions of students’ information processing. As demonstrated in the process graph, the search behavior as such cannot explain precisely why students achieved lower or higher scores. Although the number of process steps appears to be related to the task score, we need to include additional indicators of the process performance that goes along with the task performance. While all participants needed a similar amount of time to complete the task, it can be assumed that the distribution of the time spent on the individual process steps is also related to their process behavior and that it is therefore an important indicator of process performance. For instance, when students used the strategic information processing strategy instead of an avoidance strategy, the time they spent on each process step must be shorter compared to students who performed only three or four different activities. Similarly, the number of fixations while looking at different websites can provide first indications as to whether students only scrolled and quickly “skimmed” a webpage or whether they read the contents on a particular page.

To gain more insights into the task behavior and the individual process steps within the process graphs, taking into account the fixations and durations (in seconds) of the separate process steps, two students who had performed their process steps in a very typical order were selected: one from the group of students who used the “avoidance strategy” and one from the group of students who used the “strategic information processing strategy,” respectively. We selected one student with a low score and one with the high score, to investigate how and to what extent the process performance differs between two typical representatives of both groups.

**FIGURE 3:**
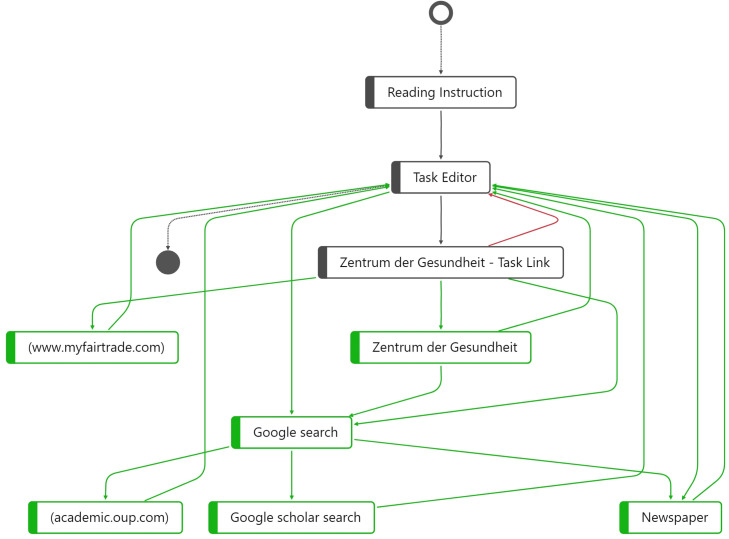
Highlighted nodes and edges for high-scoring students (green) and low-scoring student (red).

In the following section, the processes of these two students (one with a high and one with a low CORA task score) are described and compared. To this end, using the PM approach to identify similarities, differences and distinct patterns in the students’ response processes, the task-solving processes of one low-scoring student (ID 26) and one high–scoring student (ID 16) were first combined in one graph to facilitate a comparison (Figure 3).

### Distinct Process Steps

To visualize the *distinct process steps*, in Figure 3, a comparison between the two students is shown in a single process graph, which allows us to quantitatively and qualitatively describe the number and types of process steps the students performed while working on the CORA task. The process graph once again shows the differences between students who employ an avoidance strategy and those who employ a strategic information processing strategy: The colors indicate which process steps (nodes) and sequences (edges) were performed only by the low-scoring (red) or only by the high-scoring student (green). Gray nodes and edges describe process steps and edges that were the same for both students, i.e., starting the process of solving the CORA task by reading the instruction and then opening the task editor. The next process step was also identical, with both students opening the link mentioned in the task, “Zentrum der Gesundheit—Task Link,” as in the task, the respondents were asked to follow precisely this hyperlink.

From this task link onward, however, Figure 3 reveals distinctive differences between high- and low-scoring students. For instance, the low-scoring student opened the task editor again directly after accessing the task link (red edge), which indicates that the low-scoring student did not perform a web search at all—even though it was explicitly mentioned in the task prompt, again indicating the use of an avoidance strategy. In accordance with our COR construct definition, searching, evaluating, and selecting online information was considered an essential facet of COR. Without conducting a web search, it is hardly possible to evaluate the trustworthiness and reliability of online sources presented in the CORA task. This is particularly true for “Zentrum der Gesundheit—Task Link.”

The green nodes and edges show a completely different picture for the high-scoring student. After opening the task editor, the high-scoring student opened another website by “Zentrum der Gesundheit” that was not the same as the one the task link referred to, which can be considered a necessary process step to evaluate the credibility of the website as required in the task prompt. Subsequently, the high-scoring student started a Google search. The aggregated visualization in Figure 3 shows the similarities and differences in the distinct process steps, but the order of the sequences is hardly visible. Therefore, we additionally evaluated the entire underlying event log for the high-scoring student with ID 16. Regarding the order of the process steps, the data reveal the following sequences:

*(1) Reading Instruction* → *(2) Task Editor* → *(3) Zentrum der Gesundheit* → *(4) Task Link* → *(5) Google search* → *(6) (academic.oup.com)* → *(7) Task Editor* → *(8) Zentrum der Gesundheit - Task Link* → *(9) Zentrum der Gesundheit* → *(10) Google search* → *(11) Newspaper* → *(12) Task Editor* → *(13) Zentrum der Gesundheit* → *(14) Task Link* → *(15) Zentrum der Gesundheit* → *(16) Task Editor* → *(17) Newspaper* → *(18) Task Editor* → *(19) Zentrum der Gesundheit* → *(20) Task Link* → *(21) www.myfairtrade.com* → *(22) Task Editor* → *(23) Google search* → *(24) Google scholar search* → *(25) Task Editor*

This sequence of 25 process steps indicates that the first Google search was concluded by accessing the website academic.oup.com. Subsequently, the high-scoring student returned to the task editor. Following this, the task link was opened once again, followed by another webpage of “Zentrum der Gesundheit,” after which the high-scoring student conducted a second Google search. During this second Google search, the high-scoring student accessed a news site and, subsequently, returned to the task editor once again. Afterward, the high scorer accessed the news site again and then returned to the editor. The high-scoring student then conducted an additional Google search, before finally submitting the solution in the task editor. This task response behavior and these process steps can be interpreted as strategic information processing based on the definition of the COR construct measured here. The high-scoring student gathered additional information online by searching and selecting information to evaluate the reliability of the website “Zentrum der Gesundheit” before writing a response to the task.

Regarding the number of process steps, if we count the gray nodes for the low-scoring student (ID 26), the process graph reveals only three distinct process steps. In contrast, for the high-scoring student (ID 16), as shown by the gray nodes as well as the green nodes, which represent process steps unique to the high scorer, nine distinct process steps were determined. Thus, the response behaviors of low- and high-scoring student differ substantially from one another in terms of the number, kind, and order of distinct process steps.

### Fixations per Distinct Process Step

**FIGURE 4:**
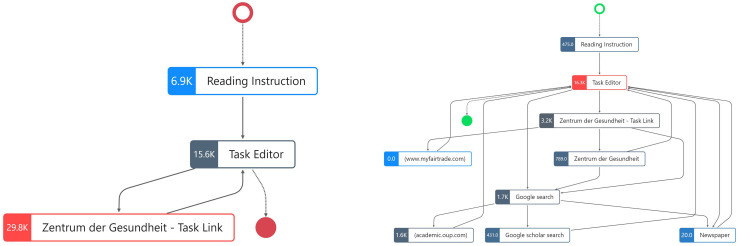
Process graph for one low-scoring student **(Left)** and one high-scoring student **(Right)** in CORA task 1 with fixations as node highlight (excluding keyboard events).

Next, to visualize the differences in the number of fixations per process step, the combined process graph from Figure 3 was split into two separate process graphs for the low- and high-scoring student (Figure 4). On the left side of Figure 4, we see the graph for the low-scoring student, and on the right side, the one for the high-scoring student. The colors of the nodes show the process steps with the highest (red) and lowest (blue) number of fixations in relation to the fixations of each student.

Comparing these two process graphs in Figure 4, it becomes evident that the high-scoring student’s fixations are distributed among eight of the nine distinct process steps, with 3,200 fixations on the website that was linked in the CORA task (“Zentrum der Gesundheit—Task Link”) and 20 to 1,600 fixations while conducting the web search. In contrast, the low-scoring student’s fixations are distributed only among the three distinct process steps, with the largest number of fixations being recorded while the low-scoring student read the task link website. However, for both students, the similar number of fixations was determined while they were actively using the task editor (15,600 for the low-scoring student and 16,300 for the high-scoring student). This means that they generated the majority of fixations while reading the task prompt and while writing their responses.

For an interpretation of when the fixations occurred within the distinct process steps such as “Task Editor,” for instance, rather while reading the task prompt or rather while writing a response, we conducted an analysis of the videos recorded by the eye tracker (Table 1). For instance, when summarizing the fixations for the process steps “Task Editor—Reading Task,” the high-scoring student read the task with 1,784 fixations, whereas the low-scoring student read it with 4,670 fixations. As the videos in combination with the event log data indicate, while the high-scoring student read the task twice (first during the initial access to the task editor and the second time after reading the “Zentrum der Gesundheit—Task Link” website and conducting the first Google search; see left side of Table 1), the low-scoring student read the task three times. The first time was also during the initial accessing of the “Task Editor,” the second time also after reading the task link website, and then the third time after he/she started to write his/her response and then returned to read the task prompt again (see right side of Table 1). This indicates that the low-scoring student based his/her response (statement on the trustworthiness of the web source) only on reading the task prompt, as well as on the task link website, which can also be considered part of the task prompt, again indicating the use of the avoidance strategy.

**TABLE 1:** Process steps and fixations in time related order of the *high* scorer (left) and the *low* scorer (right) extended by the separation in the “Task Editor” by reading and writing.

**Process step name**	**Fixations**
Reading Instruction	475
Task Editor—**Reading Task**	1,144
Zentrum der Gesundheit—Task Link	2,779
Google search	1,317
Academic.oup.com	1,582
Task Editor—**Reading Task**	640
Task Editor—**Writing Response**	1,159
Zentrum der Gesundheit—Task Link	413
Zentrum der Gesundheit	692
Google search	13
Newspaper	258
Task Editor—**Writing Response**	3,800
Zentrum der Gesundheit—Task Link	0
Zentrum der Gesundheit	97
Task Editor Reading	179
Newspaper	20
Task Editor—**Writing Response**	3,571
Zentrum der Gesundheit—Task Link	0
www.myfairtrade.com	0
Task Editor—**Writing Response**	3,021
Google search	375
Google scholar search	431
Task Editor—**Writing Response**	2,740
Reading Instruction	6,896
Task Editor—**Reading Task**	438
Zentrum der Gesundheit—Task Link	26,014
Task Editor—**Reading Task**	2,634
Task Editor—**Writing Response**	2,591
Task Editor—**Reading Task**	1,598
Task Editor—**Writing Response**	1,341
Zentrum der Gesundheit—Task Link	2,087
Task Editor—**Writing Response**	3,076
Zentrum der Gesundheit—Task Link	1,704
Task Editor—**Writing Response**	3,907

### Duration per Distinct Process Step

**FIGURE 5:**
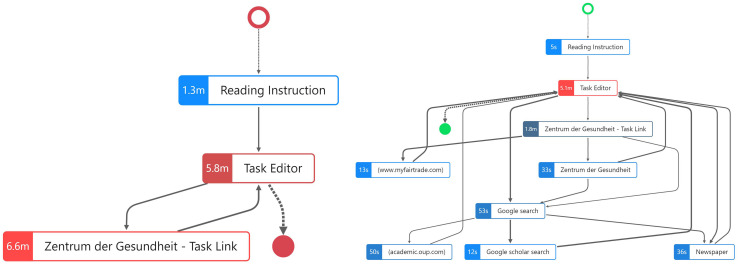
Process graph for one low-scoring student **(Left)** and one high-scoring student **(Right)** in CORA task 1 with duration as node highlight.

Regarding the duration of the identified distinct process steps (Figure 5), the low-scoring student spent more time on reading the instruction (1.3 min) compared to the high-scoring student who spent only 5 s. The low-scoring student spent the most time on reading the website linked in the task (“Zentrum der Gesundheit—Task Link” with 6.6 min), whereas the high-scoring student spent 1.8 min (Figure 5).

To conclude the PM analyses, the process performance of the high- and low-scoring student from the two groups of students using an avoidance strategy and students using strategic information processing is summarized in Table 2.

**TABLE 2:** Relevant process-related variables of the high-scoring student and the low-scoring student.

	**Low-scoring student**	**High-scoring student**
Number of process steps (full event log)	42	248
Number of distinct process steps (full event log)	10	17
Number of process steps (process mining event log)	8	22
Number of distinct process steps (process mining event log)	3	9
Average duration per step (s)	20.929	2.484
Average fixations per step	1,379.214	129.133
Score task 1	0	2

In summary, based on the PM data, it became evident that the response processes, i.e., the way high- and low-scoring students as typical representatives of the two identified response patterns process the CORA task and online information, differ substantially with regard to all process-related variables measured in this study (Table 2). These are, in particular, the total number of process steps, the distinct process steps, the number of fixations in these steps, and duration per (distinct) process step. In Table 2, we additionally distinguish between the number of (distinct) process steps between the PM data and the full event log. The full event log consists of all events that were omitted for the PM analysis (such as keyboard and mouse events, see the section “Data Transformation”). To examine whether individual differences in these response patterns can be found in the data set using the full event log, the consecutive model-based analysis building on these initial exploratory analyses was conducted (see the section “Latent Class Analyses”).

### Discussion of Process Mining Results and Research Hypotheses

To answer RQ1 (see the section “Research Objectives”), we chose a PM approach to visualize and describe the students’ response processes while solving the CORA tasks. As demonstrated in the process graphs, however, simply visualizing and exploratively analyzing the raw event log data recorded by the Tobii ET tool did not lead to a satisfying answer to RQ1. The process data were too detailed in terms of time and event granularity. Therefore, the event log data were aggregated so that the PM results led to answers in RQ1. For RQ2, which focused on finding similar and distinct patterns in the students’ responses, we were able to identify two different patterns of students’ response processes while solving the CORA tasks. In this context, we followed a stepwise approach. First, the process-related variables, including the number of fixations per individual process step, as well as the duration, number, type, and order of the individual process steps, were taken into account. Second, the task performance scores, which were performed by three independent raters, were integrated into the process data and the integrative analyses. In accordance with our COR construct definition and the theoretical assumptions (see the section “Construct Definition and Fundamental Assumptions”), the revealed patterns were defined as “avoidance strategy” vs. “strategic information processing.”

Remarkably, students from the first group show both a lower process performance and a lower task performance, in contrast to the latter group who showed a higher performance. More specifically, with regard to all assessed process-related indicators (Table 2), the students from the latter group with the higher scores process online information differently than the students from the first group with the low scores. In particular, high-scoring students process online information more intensively as indicated by a larger number of distinct process steps and total process steps, as well as more efficiently as indicated by a distribution of total fixations in these different steps, and shorter durations for each step, indicating again the use of strategic information processing according to our theoretical assumption. In contrast, the distributions of these process variables for the low-scoring students indicate the much poorer process performance as identified in the process graph for all students in this group.

To summarize the answer to RQ2, the results indicate that students with a higher process performance have significantly higher scores than students with low scores, suggesting a significant relationship between students’ process performance and their task performance. Thus, in the subsequent statistical analyses, the following two hypotheses will be tested:

*H1*: Two empirically separable student groups (high vs. low performers) can be identified based on both (i) the students’ process-related data, i.e., number and duration of the (distinct) process steps (such as searching for information, writing response) they carry out while processing the CORA tasks and the distribution of total fixations on these different steps, as well as (ii) the students’ CORA task performance (test score).

*H2*: Students who had a higher process performance, i.e., more fixations within certain (distinct) process steps and more process steps (i.e., spending less time on single task-related activities), have a higher probability to be a high performer (i.e., a higher task score), while the opposite process performance data indicate a low-performing student.

### Latent Class Analyses

To investigate the two research hypotheses H1 and H2, which are based on the empirical results to RQ2, we conducted an LCA using the same indicators as in the PM analysis and aggregating them at the student level. Before performing the latent group analyses, distributions of all assessed process-related variables [“Number of Process Steps in total”; “Number of Distinct Process Steps”; “Average Duration per Process Step” (seconds); “Average Number of Fixations per Process Step”] and the task scores, which were included in the LCA, were calculated for the entire sample (see Supplementary Figure S1).

To test *H1* and analyze whether the two distinct groups can be empirically identified among the participants, an LCA was conducted using Stata 16. The LCA classified the students with regard to both their task scores as well as the four further process-related variables concerning their entire task processing, including web search behavior (number of process steps, distinct process steps, processing duration per step, and number of fixations per step).

As the fit indices for the two-class LCA models indicate, log-likelihood, AIC, and BIC are lower in the two-class model than in the one-class model (log-likelihood with −553.371 in the one class model and −536.643 in the two-class model; AIC with 1,126.742 in the one-class model and 1,105.287 in the two-class model; BIC with 1,141.399 in the one-class model and 1,128.739 in the two-class model); all class means predicted in this LCA model are significant (Table 3). Two empirically separable groups of students (low and high performers) could be distinguished that differ significantly with regard to the measured process-related variables (process performance) and the task performance (test score).

**TABLE 3:** Predicted class means for the two groups of high- and low-performing students.

	**Group of low performers**	**Group of high performers**
*n*	21	11
No. of process steps in total	96.480***	159.407***
No. of distinct process steps	12.250***	14.591***
Duration per step (s)	9.033***	5.340***
Fixations per step	518.823***	346.352***
Score item 1	0.309**	0.882***

***p < 0.01, **p < 0.05, *p* < 0.1.

In summary, low performers perform fewer (total and distinct) process steps, spend more time on each process step, and have more fixations per process step and a lower task score. Thus, *H1* can be confirmed, indicating a significant positive relationship between the process performance and task performance in both of the two empirically distinct groups of students in this sample.

To further determine whether the differentiation between high and low performer for all participants in the sample is meaningful (H2), the posterior probability for both classes was predicted for each student based on the two-class model (Table 4).

**TABLE 4:** Variables for classification and posterior probabilities for both classes for each participant.

**Participant ID**	**Process steps**	**Distinct process steps**	**Average duration per step**	**Average fixations per step**	**Score task 1**	**Posterior probability class 2**	**Posterior probability class 1**
16	248	17	2.483871	129.1331	2	1	0
18	230	14	3.221739	256.9348	1	0.99996	0.00004
22	152	16	5.473684	262.6711	1	0.99954	0.00046
17	124	15	4.33871	341.9677	1	0.99096	0.00904
3	140	16	7.121428	517.8857	0.75	0.98744	0.01256
13	170	14	5.511765	262.3412	0.5	0.98329	0.01671
31	142	14	4.78169	378.4859	0.75	0.95424	0.04576
4	113	15	6.238938	499.6283	1	0.91722	0.08278
32	205	12	5.434146	297.5805	0.25	0.88659	0.11341
6	125	14	6.064	418.616	1	0.87108	0.12892
8	128	14	6.96875	381.4063	0.5	0.59231	0.40769
28	116	14	8.094828	353.9138	0.5	0.31101	0.68899
29	123	13	5.723577	415.0732	0.5	0.26231	0.73769
9	112	14	8.830358	613.9107	0.75	0.14116	0.85884
7	102	12	6.5	407.8235	1	0.05434	0.94566
10	91	13	9.527472	779.033	1.75	0.05142	0.94858
11	124	13	6.354839	545.9597	0	0.03728	0.96272
14	127	13	8.669291	444	0	0.02075	0.97925
19	117	13	8.717949	417.1966	0	0.01276	0.98724
5	118	12	6.779661	352.7373	0	0.00963	0.99037
30	99	11	6.242424	509.9192	1	0.00703	0.99297
15	76	14	8.947369	384.7763	0	0.00611	0.99389
1	97	13	8.329897	444.5464	0	0.00439	0.99561
12	100	11	8.86	429.89	0.5	0.00066	0.99934
23	109	11	7.651376	457.6147	0	0.00046	0.99954
21	87	12	12.2069	407.1035	0.25	0.00015	0.99985
20	68	11	8.073529	303.25	0.25	0.00013	0.99987
2	90	12	10.35556	595.9222	0	0.00011	0.99989
24	80	11	9.1	261.9375	0	0.00009	0.99991
27	72	12	9.861111	768.3333	0	0.00002	0.99998
25	59	12	10.66102	668.678	0	0.00001	0.99999
26	42	10	20.92857	1,379.214	0	0	1

As shown in Table 4, the probability of belonging to one of the two classes is higher than 70% for almost every student (see columns Posterior Probability Class 1 and 2: for class 1 participant ID’s 16–8; for class 2 participant ID’s 28–26). Each class also comprises at least one student with 100% probability (ID 26 for class 1 and ID 16 for class 2). Thus, *H2* can be confirmed.

As an additional indicator of the “process performance” of the two groups, the number of websites visited by students was analyzed. This article does not aim to analyze individually visited websites; instead, the different types of websites (e.g., newspapers, Wikipedia articles, Twitter blogs, YouTube videos) were evaluated and aggregated into meaningful categories, building distinct websites. In total, the 32 students in the sample visited 89 distinct websites for task 1. Most of these were visited by only one student (e.g., ncbi.nlm.nih.gov); only few were visited by almost all students (e.g., Google for conducting the web search). The total number of visited websites and that of distinct websites are shown in Table 5, indicating significant differences between the two groups and thus further supporting *H2*.

**TABLE 5:** Number of visited websites for high and low performers.

	**High performer (*n* = 11)**	**Low performer (*n* = 21)**
No. of distinct websites	56	33
Total no. of visited websites	265	316
Average of total no. of visited websites	24	15

## Discussion

Explorative PM provided first insights into the response processes involved in students’ CORA task solving and dealing with online information, and indicated a relationship between students’ process performance and their task performance. Existing studies already revealed differences regarding specific groups (e.g., Zhou and Ren, 2016), for instance, researchers vs. students (Wineburg and McGrew, 2017) or experts vs. novices (Brand-Gruwel et al., 2009). Based on prior research, this article distinguished groups according to a performance criterion, i.e., “process performance” and “task performance” (the CORA task score), and therefore exposes further possible distinct characteristics of response processes when dealing with online information that lead to better performance (“high performer”) or worse performance (“low performer”) in the task on COR.

Using PM analyses as an approach to visualizing and precisely describing the students’ response processes while solving the CORA tasks (RQ1), two distinct process patterns were identified among the 32 participants. In RQ2, we focused on identifying commonalities and differences in these patterns. The two identified patterns were defined as “avoidance” vs. “strategic information processing” according to our COR construct definition and the underlying theoretical framework (see the section “Theoretical Framework”). When selecting two typical representatives from the both groups, the response process of a high-scoring student (i.e., higher test score) was characterized by a higher process performance (i.e., more total process steps as well as more distinct process steps, while at the same time he/she spent less time on single process steps, e.g., on a specific webpage). Subsequently, the student from the high-scoring group distributed his/her time as well as fixations according to his/her wider range of process steps, which resulted in shorter durations per process step. In contrast, the student from the low-scoring group (i.e., lower test score) showed a lower process performance, i.e., spent most of his/her time on only one website, which led to many fixations, all of which, however, were focused on this one specific distinct process step (i.e., visiting the webpage linked in the task).

The student from the high-scoring group started writing his/her response only after conducting a web search, indicating that they weighed up different pieces of information and options, which may relate to a more analytical response process (Chen and Chaiken, 1999; Evans and Stanovich, 2013). The student from the low-scoring group started writing an answer immediately after visiting the webpage linked in the task, which indicates a tendency toward cognitive heuristics (Walthen and Burkell, 2002; Metzger, 2007) and a solution behavior characterized by using an avoidance strategy, i.e., lower cognitive effort by judging a website without searching and evaluating additional online information (as was required in the CORA task). However, these are only initial indications for a rather heuristic or strategic task processing behavior, supporting our theoretical assumptions. To be able to make more accurate statements about the actual (meta-)cognitive heuristics and information processing strategies that lead to higher vs. lower process and performance on the CORA task, a comprehensive analysis of eye movements, particularly within previously defined AOIs, would be required.

Based on the results of PM determined when answering RQ1 and the empirical findings determined when answering RQ2, two research hypotheses were formulated, and further statistical analyses were conducted to examine the response process behavior patterns in the sample. To test H1, which required us to determine each student’s probability of belonging to one of the defined subgroups of high- or low-performing students, both the process-related variables and the test score were included in an LCA. First, the sample could be divided into two distinct groups of students (“low performers” and “high performers”) by means of an LCA; here, too, the groups differed significantly in terms of task scores, as well as the process variables that had already been identified as relevant in PM, supporting two distinct process patterns: “avoidance” vs. “strategic information processing.” The LCA indicated that all of the 32 students belong to one of the two groups with a statistically high probability. As a result, H1 cannot be rejected. The results of the LCA also support that H2 cannot be rejected, as the group of high-performing students met the postulated assumptions [higher task performance (score) and higher process performance, see the section Latent Class Analyses]. However, the generalizability of these response process profiles for the overall student population requires further investigation in replication studies, including a random sampling of participants (see the sections “Discussion” and “Limitations”). It would be of particular interest to analyze in a longitudinal design how and to what extent these online information patterns may be developed over a course of study in higher education. The identified patterns in the response process behavior of students solving the CORA task should also be investigated in an experimental research design that explicitly triggers different information problem-solving strategies and (distinct) process steps (e.g., web search, evaluation of different websites) in an experimental group and with different stimuli.

In terms of contributions to the research field, our results are in line with findings from existing ET studies on web search behavior. First, as already revealed in many studies, most students have not yet developed a sufficient level of the abilities and skills (such as selecting and evaluating online information) (e.g., Walraven et al., 2009; Wineburg et al., 2018; McGrew et al., 2019) that constitute the construct of COR. Second, on the basis of ET and web search log data, we identified two groups of students who differed significantly in terms of both their test performance and all assessed response process indicators such as process steps, fixations, and duration (i.e., process performance). This finding is also in line with previous research that determined such different profiles of evaluation behavior with regard to online sources (e.g., Brand-Gruwel et al., 2017; List and Alexander, 2017).

More specifically, and also in line with previous research (e.g., Zhou and Ren, 2016), in this study, we identified substantial differences between high- and low-performing students in relation to the number, kind, and order of the distinct process steps, in particular during a web search as well as with regard to both duration and distribution of fixations per distinct process step. Using PM, we identified two very different patterns in the response processes and in particular the online search behavior of two groups of students with higher and lower CORA task scores, which were confirmed by means of an LCA. The significant differences in terms of both duration and fixations per individual step also suggest differences with regard to visual attention and eye-movement patterns between the two student groups. For instance, PM analyses indicate that students from the high-scoring group have a significantly larger number of (Google) search queries and processing activities with regard to the selected websites (reading and selecting information), indicating a strategic processing profile. In contrast, students from the low-scoring group showed only limited or even no search activity, indicating an avoidance processing profile. Combined with results regarding fixations and durations, which can be interpreted as indicators of processing new information (Holmqvist et al., 2011), these findings also initially indicate differences regarding the (meta-)cognitive activity of both student groups that need further in-depth investigation (see the section “Future Perspectives”).

### Limitations

Even though the PM analysis provided many conclusive insights into students’ task response behavior and online information processing, indicating two distinct profiles that were confirmed through LCA, it also has certain limitations. Because of the purposeful sampling based on certain defined selection criteria (Palinkas et al., 2015) but not random sampling, the representativeness of the sample is questionable, as it might affect the students’ response behavior. Thus, the generalizability of the results is limited. Moreover, as participation in this study was not mandatory and the CORA did not have any positive or negative effects on the students’ regular study progress (e.g., in the form of grades), i.e., it was a low-stakes test, the students’ motivation—which can strongly impact their task scores—is questionable.

Because we followed a person-oriented approach, variable-oriented analyses (such as regression models) were not conducted in this study. The aim was to identify subgroups and not to explain potential differences with further external criteria apart from the construct-relevant process variables. Although other contextual factors (such as the course of study and the semester) were surveyed, they were not included in the analyses so far because of the already high complexity of the analysis design. Similarly, no control was carried out on the measured personal factors (such as intelligence, expertise or previous knowledge on the topic of the CORA tasks). However, as these variables play a significant role in the handling of online information (Willoughby et al., 2009; Gadiraju et al., 2018), it cannot be ruled out that effects biased the results on COR (for implications, see the section “Latent Class Analyses”).

By using ET methodology, time-related, accurate, and exact data about the students’ solution processes were collected; however, typical ET measures were only used in a highly aggregated form for the PM analyses. High-resolution ET metrics that could have provided more detailed insights into the students’ eye movements, and therefore their gaze behavior, were not included in the PM analysis. On the one hand, this article did not focus on analyzing and interpreting ET data in terms of metrics such as fixation duration/dwell times and saccades to make inferences on visual attention and eye movements in relation to defined AOIs due to the extremely high complexity of this event log data set. On the other hand, the determination of AOI is always subject to substantial errors (Orquin et al., 2015), as it is influenced by the test designers’ opinions. Thus, in follow-up studies, an automatic determination of AOIs on the level of complete webpages will be implemented (as suggested by Hienert et al., 2019), so that an often arbitrary AOI determination cannot negatively influence the analyses.

Furthermore, as process steps were primarily analyzed quantitatively (e.g., number of total and distinct process steps), there were few qualitative differentiations between the distinct process steps, in particular regarding the qualitative characteristics of the accessed websites (such as difficulty, complexity, etc.). Nevertheless, such qualitative aspects were considered in the rating of the students’ written responses in the CORA, as one criterion for the scoring was the quality (e.g., scientific or non-scientific) of the URLs provided in the students’ responses.

Overall, the described findings emphasize the high importance of examining the processes involved in students’ ability to comprehensively deal with online information, as the level of ability to critically reflect on online information seems to be rather low among students (reflected in both the process performance and the task score distribution in this study). In this study, the students showed either an avoidance strategy or a strategic processing strategy. Although the latter led to a significantly higher CORA task performance, this strategy does not necessarily cover all main processes of COR according to our construct definition. In fact, as the distributions of the task scores indicated (see Supplementary Figure S1), only two students from this group achieved the maximum score of two points. Hence, further research is required to understand and explain processes that lead to this low COR skill level among many students and, consequently, to deduce how a critically reflective handling of online information could be promoted in higher education.

### Future Perspectives

In the next research step, a more in-depth qualitative analysis of the identified groups and response process patterns would be required to build a solid basis for the formulation of hypotheses with regard to theoretically expected gaze patterns (Pifarré et al., 2018). For further studies, therefore, the students’ (meta-)cognitive processes when dealing with online information should be investigated in more depth and in a longitudinal design. Experimental between-subject design, for instance, regarding the search behavior with and without prior instruction, could be implemented here. Eye-movement diagnostics should also be brought more into focus to enable more specific descriptions of students’ visual attention and indications of cognitive load.

The person-oriented methodological approach applied here should be expanded and combined with a variable-oriented approach to take into account any contextual or personal factors that may influence gaze behavior (such as complexity of presented information and general cognitive ability), as many studies indicate (Horstmann et al., 2009; Raney et al., 2014). In subsequent studies, therefore, potential explanatory variables of the various processes must also be included in a variable-oriented approach to test for discriminant validity as well. For instance, the question arises as to whether different response processes are to be expected among students with a certain academic subject, certain educational indicators, or in different familial and social contexts. In particular, the possible effects of different domains and possible curricular and/or instructional specifics in the study programs that may impact students’ COR should also be considered in follow-up research to test for instructional validity (Pellegrino et al., 2016).

Qualitative studies, for instance, rating the different websites used by each student, need to be conducted as well to enable further qualitative analyses of the sequences of information processing. Because of the open nature of the CORA, the primary question is how to find the best way to deal with the task, which is particularly useful for instruction and teaching in higher education. For example, the question is whether a certain process step or a sequence of process steps is decisive for a successful solution of the CORA task. In the past, there have been frequent studies on backward or forward reasoning, which find ideal task solvers in various test environments (Norman et al., 1999). A similar question needs to be researched on the basis of the available findings: can ideal solution patterns be found in (partial) sequences, and can instructional settings be developed based on these patterns, for instance, by indicating to the learner that a text should be read first, and the web search carried out subsequently and with a certain term specification? Additional explanations by the test takers, for instance, through concurrent verbal protocols, could provide further insights into the students’ causal decision contexts (Leighton and Gierl, 2007) and be combined and evaluated in parallel to the time-sequential recordings of the ET data (Maddox et al., 2018) and predefined process steps.

In this study, we used the purposeful sampling method (Palinkas et al., 2015) and focus on specific characteristics in our sampling to identify differences in the construct and the students’ processes while responding to the CORA tasks. Using this sampling approach, we identified distal indicators to analyze the breadth of possible response processes. However, the criteria for purposeful sampling can be expanded in future studies, to, e.g., include other indicators such as intelligence or domain-specific prior knowledge. The effects of these kinds of additional indicators on COR processes need to be sufficiently analyzed in follow-up research. This should include adding further domains in replication studies.

Overall, it would be of great value for further experimental and longitudinal studies to consider the students’ handling of online information in a differentiated way with regard to additional contextual factors and personal factors (at different levels of analysis) to control for intercorrelations, in an integrated person-variable–oriented approach (Rauthmann and Sherman, 2016).

## Data Availability Statement

The raw data supporting the conclusions of this article will be made available by the authors, without undue reservation.

## Ethics Statement

The studies involving human participants were reviewed and approved by the State Officer for Data Protection and Freedom of Information Rhineland-Palatinate. The participants provided their written informed consent to participate in this study.

## Author Contributions

SS co-developed the assessment, conducted the analyses, and co-wrote the manuscript. OZ-T provided the idea for the study, co-developed the assessment, supervised the analyses, and co-wrote the manuscript. JR was involved in the data collection and in preparing and reviewing the manuscript. VK and MW were involved in the data collection, and supported the analyses. A-KB co-implemented the ET test environment, and was involved in the data collection, and in preparing the manuscript, and supported the analyses. SB co-developed the ET test environment and was involved in the analyses, and in preparing the manuscript. All authors contributed to the article and approved the submitted version.

## Conflict of Interest

The authors declare that the research was conducted in the absence of any commercial or financial relationships that could be construed as a potential conflict of interest.

## Footnotes

1 https://edupad.ch/#about

2 http://www.pafnow.com/

3 https://www.stata.com/
