# Supplementary figures and images for: Undergraduate Students’ Critical Online Reasoning—Process Mining Analysis
Source: Front Psychol. 2020 Nov 30;11:576273. doi: 10.3389/fpsyg.2020.576273 (PMC7793971; doi:10.3389/fpsyg.2020.576273)

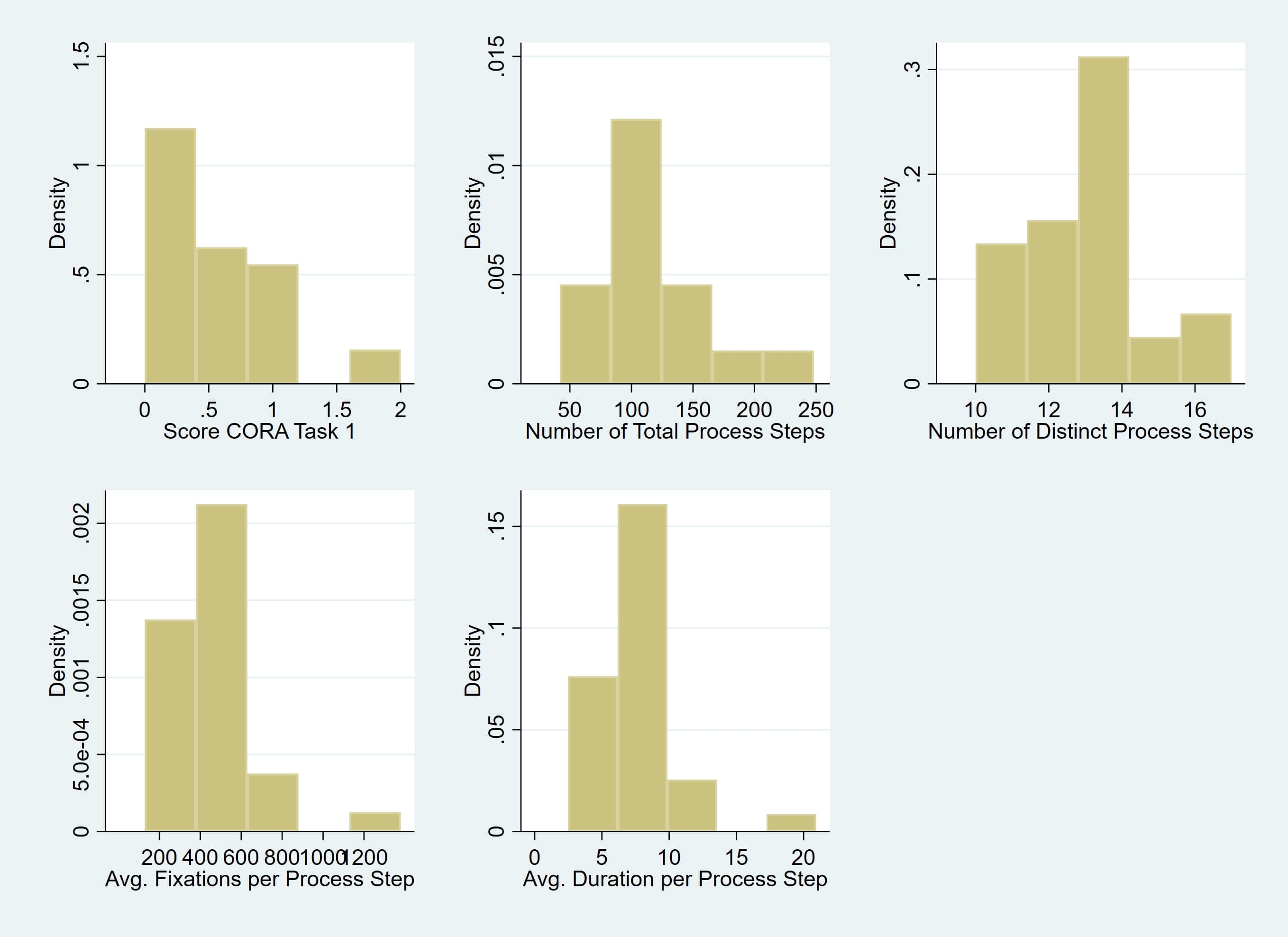

Supplement: Supplementary file 1 [file Image_1.jpg]

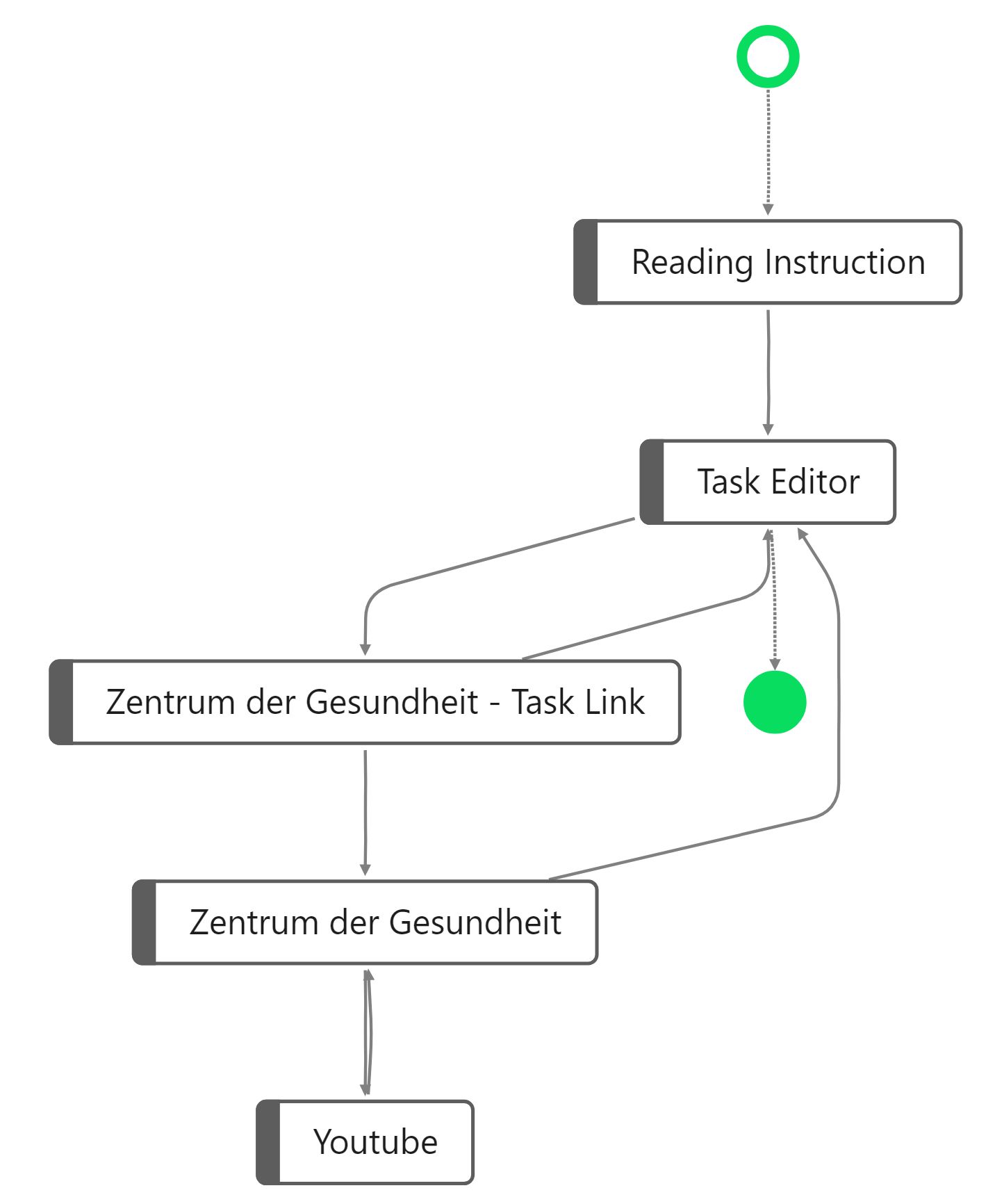

Supplement: Supplementary file 2 [file Image_2.jpg]

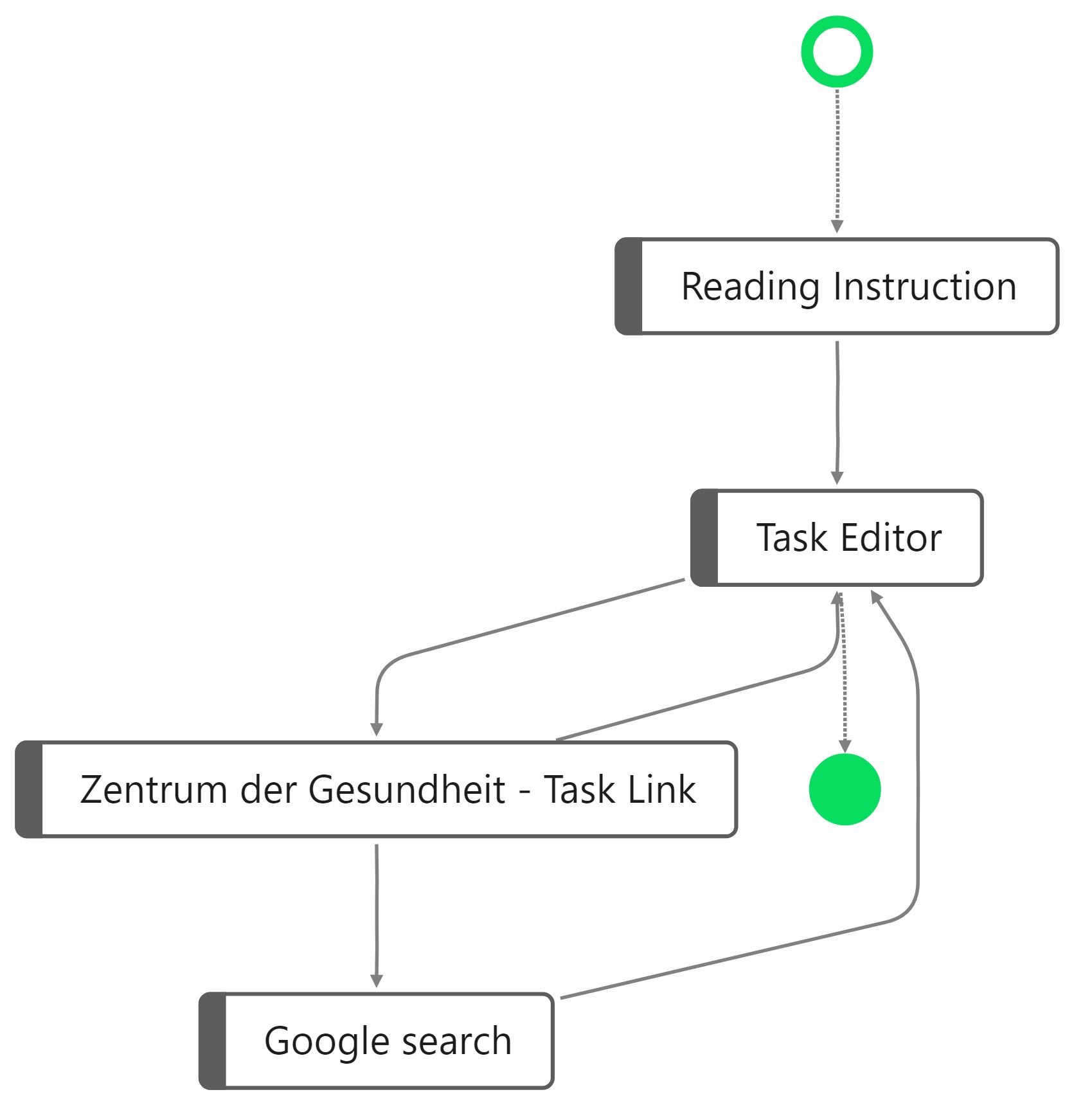

Supplement: Supplementary file 3 [file Image_3.jpg]
