# Supplementary material for: Undergraduate Students’ Critical Online Reasoning—Process Mining Analysis
Source: Front Psychol. 2020 Nov 30;11:576273. doi: 10.3389/fpsyg.2020.576273 (PMC7793971; doi:10.3389/fpsyg.2020.576273)
Supplement: Supplementary file 4 [file Table_1.DOCX]

Supplementary Material

# Supplementary Figures and Tables

## Supplementary Tables

Supplementary Table 1. Example excerpts from Tobii raw event log data for the sequence of a web page visit for one student (rows in between with null values omitted for clarity).

| **Recording Timestamp** | **Event** | **Event value** | **Presented Stimulus name** | **Eye movement type** |
| --- | --- | --- | --- | --- |
| 248384426 | URLStart | Veganes Eiweiss – Pflanzliche Proteinquellen (www.zentrum-der-gesundheit.de) | NULL | Saccade |
| 248388985 | NULL | NULL | Web Page | Saccade |
| 248397339 | NULL | NULL | Web Page | Saccade |
| 283672574 | KeyboardEvent | [Ctrl] + v | NULL | Fixation |
| 283672574 | NULL | NULL | Web Page | Fixation |
| 283675791 | NULL | NULL | Web Page | Fixation |
| 283684145 | NULL | NULL | Web Page | Fixation |
| 283784106 | NULL | NULL | Web Page | Fixation |
| 283791755 | URLEnd | Veganes Eiweiss – Pflanzliche Proteinquellen (www.zentrum-der-gesundheit.de) | NULL | Fixation |
| 283792459 | NULL | NULL | Web Page | Fixation |
| 283800772 | NULL | NULL | Web Page | Fixation |
| 283809104 | NULL | NULL | Web Page | Fixation |
| 283817445 | NULL | NULL | Web Page | Fixation |
| 283825769 | NULL | NULL | Web Page | Fixation |
| 283834112 | NULL | NULL | Web Page | Fixation |
| 283836244 | URLStart | Zentrum der Gesundheit - Das ganzheitliche Gesundheitsportal (www.zentrum-der-gesundheit.de) | NULL | Fixation |
| 283842565 | NULL | NULL | Web Page | Fixation |

Supplementary Table 2. Examples of visited websites from complete sample.

| **Long URL Link** | **Short Webpage name for Analyses** |
| --- | --- |
| Health effects of vegan diets \| The American Journal of Clinical Nutrition \| Oxford Academic (academic.oup.com) | (academic.oup.com) |
| Wie pruefe ich die Seriositaet von Quellen im Internet? - www.planet-beruf.de (planet-beruf.de) | (planet-beruf.de) |
| Liste veganer Proteinquellen - Übersicht der Lebenmittel (vegane-proteinquellen.de) | (vegane-proteinquellen.de) |
| So erkennst du seriöse Quellen im Internet für deine Bachelorarbeit (www.korrektur-plus-lektorat.de) | (www.korrektur-plus-lektorat.de) |
| Natürliche Nahrungsergänzungsmittel \| myFairtrade (www.myfairtrade.com) | (www.myfairtrade.com) |
| Comparison of Nutritional Quality of the Vegan, Vegetarian, Semi-Vegetarian, Pesco-Vegetarian and Omnivorous Diet (www.ncbi.nlm.nih.gov) | (www.ncbi.nlm.nih.gov) |
| Vegane Ernährung – Die wichtigsten Nährstoffe auf einen Blick (www.peta.de) | (www.peta.de) |
| myfairtrade.com Erfahrungen \| Lesen Sie 26.296 myfairtrade.com Bewertungen (www.trustedshops.de) | (www.trustedshops.de) |
| Google Scholar (scholar.google.de) | Google scholar search |
| Clarys P, Mullie P et al.,Comparison of Nutritional Quality - Google-Suche (www.google.de) | Google search |
| Craig WJ, Health effects of vegan diets, American Journal of Clinical Nutrition - Google-Suche (www.google.de) | Google search |
| dr. med jochen handel - Google-Suche (www.google.de) | Google search |
| Google (www.google.de) | Google search |
| my fairtrade seriös - Google-Suche (www.google.de) | Google search |
| Neosmart Consulting AG - Google-Suche (www.google.de) | Google search |
| wann ist eine Quelle seriös - Google-Suche (www.google.de) | Google search |
| zuverlässige internetquellen - Google-Suche (www.google.de) | Google search |
| Das Online-Geschäft mit dubiosen Gesundheitstipps - Berliner Morgenpost (www.morgenpost.de) | Newspaper |
| Das Online-Geschäft mit dubiosen Gesundheitstipps - Hamburger Abendblatt (www.abendblatt.de) | Newspaper |
| DER SPIEGEL \| Online-Nachrichten (www.spiegel.de) | Newspaper |
| Pflanzliche oder tierische Proteine: Langzeitstudie belegt es – darum leben Veganer gesünder als Fleischesser \| Berliner Zeitung (archiv.berliner-zeitung.de) | Newspaper |
| Protein: Pflanzliches Eiweiß gesünder als tierisches - DER SPIEGEL (www.spiegel.de) | Newspaper |
| Wundermittel: Die Fallen im Internet \| ZEIT ONLINE (www.zeit.de) | Newspaper |
| EduPad \| 1.7.5 (edupad.ch) | Task Editor |
| YouTube (www.youtube.com) | Youtube |
| ZDG Portrait: Ben – Deutsch - YouTube (www.zentrum-der-gesundheit.de) | Youtube |
| Zentrum der Gesundheit - YouTube (www.youtube.com) | Youtube |
| Alles durchsuchen (www.zentrum-der-gesundheit.de) | Zentrum der Gesundheit |
| Fleisch als Überträger des Coronavirus (www.zentrum-der-gesundheit.de) | Zentrum der Gesundheit |
| Proteinbedarf vegan decken – vegane Proteine (www.zentrum-der-gesundheit.de) | Zentrum der Gesundheit |
| Themen von A-Z (www.zentrum-der-gesundheit.de) | Zentrum der Gesundheit |
| Zentrum der Gesundheit - Das ganzheitliche Gesundheitsportal (www.zentrum-der-gesundheit.de) | Zentrum der Gesundheit |
| Veganes Eiweiss – Pflanzliche Proteinquellen (www.zentrum-der-gesundheit.de) | Zentrum der Gesundheit - Task Link |

Supplementary Table 3. Example excerpt of full event log for LCA.

| **CaseId** | **ActivityName** | **Timestamp** | **TimestampEnd** | **Fixations** |
| --- | --- | --- | --- | --- |
| 8 | Recording Start | 2020-02-03 12:36:05.7566667 | 2020-02-03 12:36:06.7766667 | 0 |
| 8 | Eyetracker Calibration | 2020-02-03 12:36:06.7766667 | 2020-02-03 12:37:26.9133333 | 5,324 |
| 8 | MouseEvent | 2020-02-03 12:37:26.9133333 | 2020-02-03 12:37:26.9133333 | 0 |
| 8 | Eyetracker Calibration | 2020-02-03 12:37:26.9666667 | 2020-02-03 12:38:14.1266667 | 3,259 |
| 8 | Reading Instruction | 2020-02-03 12:38:14.1466667 | 2020-02-03 12:39:03.3933333 | 739 |
| 8 | StartTask | 2020-02-03 12:39:03.3966667 | 2020-02-03 12:39:03.4300010 | 0 |
| 8 | WebStimulusStart | 2020-02-03 12:39:03.4800000 | 2020-02-03 12:39:03.7033333 | 0 |
| 8 | Task Editor | 2020-02-03 12:39:03.7066667 | 2020-02-03 12:39:37.4433333 | 2,187 |
| 8 | Mouse Event | 2020-02-03 12:39:37.4433343 | 2020-02-03 12:39:37.4433353 | 1 |
| (…) |  |  |  |  |
| 8 | Task Editor | 2020-02-03 12:44:17.4433353 | 2020-02-03 12:45:17.1600000 | 2,743 |
| 8 | WebStimulusEnd | 2020-02-03 12:45:17.1600000 | 2020-02-03 12:45:17.1600000 | 0 |
| 8 | WebStimulusStart | 2020-02-03 12:45:16.1300000 | 2020-02-03 12:45:16.3866667 | 6 |
| 8 | Zentrum der Gesundheit - Task Link | 2020-02-03 12:45:16.3933333 | 2020-02-03 12:45:20.8300000 | 476 |
| 8 | MouseEvent | 2020-02-03 12:45:22.5633343 | 2020-02-03 12:45:22.8333333 | 0 |
| 8 | KeyboardEvent - Other | 2020-02-03 12:45:22.8366667 | 2020-02-03 12:45:23.1066667 | 5 |
| 8 | Zentrum der Gesundheit - Task Link | 2020-02-03 12:45:23.1066667 | 2020-02-03 12:45:34.9000000 | 340 |
| 8 | WebStimulusEnd | 2020-02-03 12:45:34.9000000 | 2020-02-03 12:45:34.9000000 | 0 |
| 8 | WebStimulusStart | 2020-02-03 12:45:34.9000010 | 2020-02-03 12:45:35.1700000 | 0 |
| 8 | Task Editor | 2020-02-03 12:45:35.1733333 | 2020-02-03 12:45:42.3666667 | 333 |
| 8 | KeyboardEvent - Other | 2020-02-03 12:45:42.3666667 | 2020-02-03 12:45:42.6300000 | 0 |
| 8 | KeyboardEvent - Writing |  |  |  |
| (…) |  |  |  |  |

Supplementary Table 4. Example of short event log for process mining analysis.

| **CaseId** | **ActivityName** | **Timestamp** | **TimestampEnd** | **Fixations** |
| --- | --- | --- | --- | --- |
| 8 | Reading Instruction | 2020-02-03 12:38:14.1466667 | 2020-02-03 12:39:03.3933333 | 739 |
| 8 | Task Editor | 2020-02-03 12:39:03.7066667 | 2020-02-03 12:45:17.1600000 | 4,931 |
| 8 | Zentrum der Gesundheit - Task Link | 2020-02-03 12:45:16.3933333 | 2020-02-03 12:45:34.9000000 | 821 |
| 8 | Task Editor | 2020-02-03 12:45:35.1733333 | 2020-02-03 12:45:42.6300000 | 333 |
| (…) |  |  |  |  |

## Supplementary Figures

**Supplementary Figure 1.** Histogram for all aggregated process-relevant variables on the student level.

**Supplementary Figure 2.** A process variant for a student using an avoidance strategy (Youtube).

**Supplementary Figure 3.** A process variant for a student using an avoidance strategy (Google).
